# Millimeter-Wave Directed Energy-Mediated Neural Cell Injury: Insights into Protein Degradation and Cell Injury Mechanisms

**DOI:** 10.21203/rs.3.rs-7367389/v1

**Published:** 2025-09-05

**Authors:** Mojtaba Golpich, Jiepei Zhu, Firas Kobeissy, Kevin K. Wang, Hamad Yadikar, Sherifdeen Onigbinde, Joy Solomon, Vishal Sandilya, Moyinoluwa Adeniyi, Yehia Mechref, Firas Kobeissy, Kevin K. Wang

**Affiliations:** Center for Neurotrauma, Multiomics & Biomarkers (CNMB), Department of Neurobiology & Neuroscience Institute, Morehouse School of Medicine, Atlanta, GA 30310, USA; Department of Biological Sciences, Faculty of Science, Kuwait University, Sabah Al Salem University City, Kuwait City 13060, Kuwait; OMICS Research Unit, Research Core Facility, Faculty of Medicine, Kuwait University, Kuwait City 13110, Kuwait; Chemistry and Biochemistry Department, Texas Tech University, Lubbock, TX 79409, USA; Center for Visual & Neurocognitive Rehabilitation (CVNR), Atlanta VA Health Care System 1670 Clairmont Rd, Decatur, GA 30033, USA

**Keywords:** Millimeter-Wave Directed Energy, Cell Viability, Cell Membrane Integrity, Cell Death, Protein Degradation

## Abstract

**Background:**

Millimeter-wave directed energy (mmWave DE) is increasingly used in telecommunications and military applications, yet its biological effects on neuronal systems remain unclear. To characterize the spatially dependent cellular and molecular impacts of 34.14 GHz mmWave DE exposure on mouse neuroblastoma (N2A) cells as a model system.

**Methods:**

N2A cells were exposed to mmWave DE for 48 hours, generating three defined zones: Center (direct exposure), Penumbra (peripheral exposure), and Control. Morphological changes were evaluated by microscopy; viability and membrane integrity were assessed via MTT and LDH assays. Cytoskeletal protein degradation (αII-Spectrin, Vimentin) was measured by Western blot. Label-free LC-MS/MS proteomics, Gene Ontology (GO), Ingenuity Pathway Analysis (IPA), and Pathway Studio were used to define global molecular responses.

**Results:**

Center-exposed cells showed severe morphological disruption, reduced viability (*p* < 0.0001), and elevated LDH release, consistent with necrosis. Western blot revealed increased proteolysis of αII-Spectrin and Vimentin. Proteomic profiling identified >180 dysregulated proteins in the Center and 34 in the Penumbra, affecting cytoskeleton organization, mitochondrial function, and RNA processing. GO and IPA indicated activation of apoptosis, ER stress, and necrosis pathways, with inhibition of translation and cell movement. Pathway mapping linked DE-altered proteins to neurodegenerative and injury-relevant processes.

**Conclusion:**

mmWave DE exposure induces graded cellular injury, ranging from stress adaptation in peripheral regions to proteostasis collapse and structural failure in direct-hit zones. These findings support reconsideration of mmWave safety standards and highlight parallels with neurodegenerative mechanisms.

## Introduction

Over the past three decades, the proliferation of electronic devices such as cell phones, communication towers, high-voltage power lines, various electronic tools, and microwave ovens has significantly increased. This rise in usage has led to what is often referred to as “electromagnetic smog,” a byproduct of the electromagnetic radiation (EMR) these devices emit. The emission of radio frequency-electromagnetic radiation (RF-EMR) from widely used devices has emerged as a public health concern due to its harmful effects (1). The increasing prevalence of human exposure to electromagnetic fields (EMF) from various sources in everyday life has raised concerns about potential health implications (2). Among these, man-made static fields are commonly encountered in occupational settings, such as near magnetic resonance imaging (MRI) scanners or Fifth-generation (5G) wireless stations (3, 4). The millimeter-wave (mmWave) EMR is a band of radio frequency (RF) energy in the electromagnetic spectrum that varies from 30 to 300 GHz with the wavelengths ranging from 10 millimeters down to 1 millimeter, depending on their specific frequency within the given range (5). It has emerged as a promising technology with diverse applications, ranging from telecommunications, non-lethal crowd control systems, security/military applications, and surveillance technologies (6). However, concerns regarding the potential adverse effects of mmWave exposure on biological tissues, particularly the brain (7), have prompted extensive research in this area. Notably, mmWave EMR exposure has been associated with DNA damage and alterations in the central nervous system (1).

The millimeter-wave directed energy (mmWave DE) is a type of directed energy weapon (DEW), also known as RF weapons, that uses high-frequency EMR, such as microwaves, to disable electronic systems. Detection of DE exposure is challenging, as it is non-invasive and involves minimal physical contact with subjects. The increasing use of DEWs is a growing threat to military forces on the battlefield (8, 9). The use of RF energy as a tool of espionage or weaponry by U.S. adversaries has raised security concerns. Recently, a neurological condition known as Havana syndrome has gained attention, characterized by a set of acute symptoms reported by US personnel in Havana, Cuba, and other locations (10). Investigations by Academies of Sciences, Engineering, and Medicine have linked this syndrome to the effects of directed, pulsed radio frequency (microwave) energy, which is used to mean EMR with wavelengths ranging from about one meter to one millimeter, corresponding to frequencies between 300 MHz and 300 GHz, respectively. Notably, mmWave energy in the 30–300 GHz range, which is known as the extremely high frequency band, has emerged as a potential cause for the syndrome, owing to its higher frequency allowing for more directional propagation and possible weaponization (11, 12, 13, 14). Many *in vitro* studies have been done to investigate the effects of mmWave DE. Li et al. (2021) showed that exposure to RF-EMR impairs neurite outgrowth in primary mouse hippocampal neurons and Neuro2a cells. This RF-EMR exposure deformed neuronal cell morphology, which may also cause neurodegenerative diseases such as Alzheimer’s disease and Parkinson’s disease (15). Zymantiene et al. (2020) demonstrated that RF exposure caused shrinkage of pyramidal neurons, perineuronal edema, and vacuolization in cortical neurons and astrocytes (16). Although many studies have been conducted on the effects of mmWave DE, there is a lack of reliable bioeffects studies to understand the potential biological effects of exposure to high-frequency (24–47 GHz) RF signals (17). Given the evolving nature of mmWave DE and its possible implications for neurological health, it is essential to further investigate its impact on neuronal cells and cellular responses.

In the present study, we chose to expose the cells to a selected mmWave DE with a frequency of 30 GHz, as it falls within the range of the frequency band used for 5G wireless communication and radar systems (18). It could also be postulated as a better mimic of Havana syndrome and is expected to cause more brain damage. This study aims to contribute to our understanding of the effects of mmWave DE exposure on neuronal cells, focusing on structure, function, and cellular changes associated with protein degradation and cell death pathways. The findings from this research may provide critical insights into the potential diagnostic and prognostic biomarkers for assessing the damage effects of DE exposure, ultimately contributing to the development of practical safety guidelines and protective measures. Therefore, developing, refining, and examining the modeling and understanding of the underlying mechanisms of mmWave DE-mediated neuronal injury is crucial for the safe and responsible application of this technology.

## Materials and methods

### Cell culture and treatment

The Neuro2a (N2a) cells, a fast-growing mouse neuroblastoma cell line (CCL-131), were obtained from the American Type Culture Collection (ATCC, USA). N2A cells were cultured in two 12-well plates at a density of 2.5 × 10^5 cells/mL/well using 1 mL of DMEM medium (Sigma-Aldrich, USA) supplemented with 10% (v/v) heat-inactivated fetal bovine serum (FBS; Sigma, USA), L-glutamine (2 mM), and sodium pyruvate (1 mM), 1% penicillin–streptomycin (Sigma-Aldrich, USA), and 1% Fungizone (amphotericin B; Sigma-Aldrich, USA) (1 mL culture medium/well) and incubated at a temperature of 37°C with 5% CO_2_ and 95% air humidity.

### Experimental design

**Figure 1:**
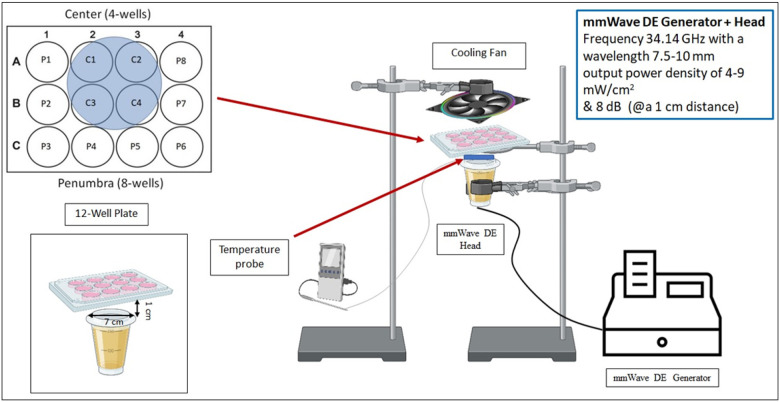
Schematic illustration of the experimental design. In this study, N2A cells were cultured in a 12-well plate and exposed to bottom-up mmWave DE irradiation (30 min exposure and 15 min rest, repeated five times over two consecutive days), generated by a mmWave DE generator. The DE is radiated to the cells by the head of the device (diameter: 7 cm), 1 cm from the bottom of the plate. To prevent the increase in heat caused by the exposure of waves on top of the plate, a cooling fan was used. During the test, the temperature of the plate surface was monitored with a thermometer. P, Penumbra; C, Center.

The first plate was assigned to the cells exposed to the mmWave DE irradiation, which were used as the center (n = 4) and penumbra (n = 8) groups, while the second plate was allocated to the non-exposed cells that were used as the control group (n = 6). All experiments were performed in triplicate. The experimental set-up used to generate mmWave DE irradiations consisted only of an electromagnetic wave generator (GradyVet MilliWave, USA) working in the frequency range of 30–45 GHz (wavelength: 7.5–10 mm & output power density: 4–9 mW/cm^2^). We set an output power of 8 dB (mW) corresponding to a frequency of 34.14 GHz. Before irradiating the sub-confluent cells grown in 12-well plates, their culture medium is discarded, the cells are washed with 37°C Phosphate-buffered saline (PBS) buffer (Sigma-Aldrich, USA), and then they are replaced with the culture medium without FBS (37°C). Then, sub-confluent cells grown in a 12-well plate were exposed to the mmWave DE irradiation with a frequency of 34.14 GHz through the electromagnetic wave generator at room temperature. In this study, cells were exposed to the bottom-up mmWave DE irradiation (30 min exposure and 15 min rest, 5 times/day) for 48 hours (h). As shown in Fig. 1, the mmWave irradiation head was positioned at 1 cm from the bottom of the plates containing the cells. This distance was necessary to avoid the heat produced by the device. Additionally, a cooling fan was used above the plates during the experiment to prevent the temperature of the cell culture medium from increasing due to radiation. To verify whether the electromagnetic wave generator heated the sample, we carried out temperature measurements by using a digital thermometer (Taylor 9940, China). The temperature probe was positioned inside the sample under the mmWave irradiation head. The measurements were conducted with the generator turned off and on for a duration of 4 h. In both cases, there was no increase in sample temperature. Based on the surface area of the mmWave irradiation head and the area covered by its radiation directly and indirectly on the plates, the irradiated cells were divided into center (A2, A3, B2, B3) and penumbra (A1, A4, B1, B4, C1, C2, C3, C4) groups (Fig. 1).

### Morphological assessment

Morphological evaluation of cells was done for cells seeded in 12-well plates (2.5 × 10^5 cells/well) 48 h after the last exposure. An inverted phase-contrast light microscope (Olympus, Japan) was used to capture microscopic images, allowing for the observation and evaluation of morphological changes in cells exposed to mmWave DE irradiation. Each representative image was selected and photographed for each group from a minimum of three randomly selected microscopic fields (magnification, 250×). All experiments were repeated at least three times.

### MTT assay

The cell viability of N2A cells exposed to the mmWave DE irradiation was evaluated using the MTT (3-(4,5-dimethylthiazol-2-yl)-2,5-diphenyltetrazolium bromide) assay. This assay is based on the conversion of the water-soluble MTT reagent to an insoluble formazan product in the presence of redox potential in viable mammalian cells. 48 h after the cells were exposed to the last exposure, the cells were washed by removing the medium, using PBS, and replacing it with 500 μL of fresh medium/well. Then, 50 μL of the 12 mM MTT stock solution was added to each well and incubated at 37°C (5% CO_2_) in the dark for 4 h. After incubation, 500 μL of the SDS (sodium dodecyl sulfate)-HCl (hydrochloric acid) solution (1 mg of the SDS + 10 mL of the 0.01 M HCl) was added to each well and incubated (37°C, 5% CO_2_) for another 4 h. Mix each sample by pipetting up and down to dissolve the precipitated formazan crystals in SDS-HCl solution. The solubilization of the formazan crystals by the SDS reagent results in a change in absorbance. Finally, the absorbance of each well was measured using a SpectraMax iD3 microplate reader (Molecular Devices, USA) at a wavelength of 570 nm. All experiments were repeated at least three times. The results were shown as the percentage of cell death (cytotoxicity). According to the following equation, the cell viability (%) was calculated by a percentage relative to the control cells and expressed as means ± standard error of the mean (SEM):

$$\text{Cell}\;\text{Viability}\;\left(\%\right)=\frac{\left(\text{OD}\;\text{Sample}\;-\;\text{OD}\;\text{Blank}\right)}{\left(\text{OD}\;\text{Control}\;-\;\text{OD}\;\text{Blank}\right)}\times 100$$

### LDH release assay

Lactate dehydrogenase (LDH) release assay, a quantitative colorimetric method, was used to determine cytotoxicity. Specifically, the assay can help assess whether exposure to mmWave DE irradiation causes damage to cell membrane integrity, leading to cell death and leakage of the LDH enzyme into the culture medium. 48 hours after the cells were exposed to the last treatment, the culture medium was aspirated and centrifuged for 5 minutes at 3000 rpm to obtain a cell-free supernatant. LDH activity in the medium was determined using a commercially available kit (Promega, USA). This assay is based on the conversion of lactate to pyruvate in the presence of LDH, accompanied by a parallel reduction of NAD. The formation of NADH from the above reaction results in a change in absorbance at 490 nm. 50 μL of culture medium and the same amount of warm CytoTox 96 reagent were mixed in a 96-well plate (Thermo Scientific, USA). After 30 minutes of incubation at room temperature in the dark, 50 μL of stop solution was added to the reactions. The absorbance was then recorded at 490 nm using a SpectraMax iD3 microplate reader (Molecular Devices, USA). All experiments were repeated at least three times. According to the following equation, the cell death ratio was calculated relative to the control cells and expressed as means ± SEM.

$$\text{Cell}\;\text{Death Ratio}\;=\frac{\left(\text{OD}\;\text{Sample}\;-\;\text{OD}\;\text{Blank}\right)}{\left(\text{OD}\;\text{Control}\;-\;\text{OD}\;\text{Blank}\right)}$$

### Western blotting

Expression of proteins associated with cell membrane integrity, including αII-Spectrin and Vimentin, in control and DE-exposed cells was evaluated by Western blot. Cells were washed three times with ice-cold PBS to remove residual media and cellular debris. For protein extraction, cells were lysed using Triton X-100 cell lysis buffer to ensure efficient cellular protein recovery while maintaining protein integrity. The lysis buffer composition was as follows: 50 mM Tris-HCl (pH 7.4), 150 mM NaCl, 1% Triton X-100, supplemented with a protease inhibitor cocktail (1 tablet/10 mL; Millipore Sigma, USA) and 1% phosphatase inhibitor cocktail (Sigma-Aldrich, USA). Approximately 0.2 mL of lysis buffer was used per 10^6 cells. Cells were scraped off the dish and then incubated on ice while shaking for one hour to ensure complete lysis. The lysates were transferred to a microcentrifuge tube and then centrifuged at 13,000 rpm for 15 minutes at 4°C. The supernatant, containing the soluble protein fraction, was carefully transferred to a new tube. Protein concentration in the lysates was determined using the Bradford Protein Assay (Bio-Rad Laboratories, USA). Briefly, 5 μL of each sample was mixed with 225 μL of Bradford reagent and incubated at room temperature for 15 minutes. The absorbance at 750 nm was measured using a spectrophotometer. A standard curve was prepared using known concentrations of bovine serum albumin (BSA) to calculate the protein concentrations of the samples. An aliquot containing 20 μg of total protein from each sample was prepared with PBS and 4x Laemmli sample buffer (Bio-Rad Laboratories, USA), then denatured by heating at 95–100°C for 5 min and loaded (20 μg/20 μL per well) on a 4–20% tris-glycine mini protein gel (Invitrogen, USA). To facilitate the separation of proteins based on their molecular weight, a sodium dodecyl-sulfate polyacrylamide gel electrophoresis (SDS-PAGE) was used. Following electrophoresis, proteins were transferred onto polyvinylidene difluoride (PVDF) membranes (Invitrogen, USA) using the iBlot gel transfer device (Invitrogen, USA). The PVDF membranes were then blocked with a solution containing 1x Tris-buffered saline, 0.1% Tween 20 (TBST) buffer (Invitrogen, USA), and 5% non-fat milk to prevent non-specific antibody binding. Incubation with primary antibodies including anti-αII-Spectrin mAb antibody (Cat#: BML-FG6090-0500, Enzo Life Sciences, USA), anti-Vimentin mAb antibody (Cat#: ab92547, Abcam, USA), and anti-β-actin mAb antibody (Cat#: A5441, Sigma-Aldrich, USA) allowed for the specific detection of the targeted proteins, while subsequent incubation with secondary antibodies including alkaline phosphatase (AP)-conjugated goat anti-mouse & -rabbit IgG (Cat#: 115-055-062 & 111-055-045, Jackson Immuno Research, USA). It was followed by BCIP/NBT 1-component phosphatase substrate (Cera Care, USA) that facilitated the visualization of the protein bands. Protein quantification was performed by ImageJ 1.54g (Java 1.8.0). The original uncropped immunoblot and Coomassie-stained gel images are shown in **Supplementary Fig. 1**.

### Quantitative proteomics by LC-MS/MS

A label-free quantitative proteomics approach was employed to profile global protein expression changes in the Control, Center, and Penumbra groups. This involved the tryptic digestion of cell lysate proteins and analysis by nano-liquid chromatography-tandem mass spectrometry (LC-MS/MS), followed by label-free quantification.

### Extraction and tryptic digestion of proteins

To do this, we extracted the protein from the cell pellets and measured their concentrations as described in the previous section. Extracted proteins (10 μg from each sample) were first denatured at 80°C for 10 minutes, followed by incubation with 200 mM dithiothreitol (DTT) at 60°C for 45 minutes. After reduction, proteins were alkylated by adding 2–6μl of a 200 mM Iodoacetamide (IAA) solution and incubating in the dark at 37°C for 45 minutes. Any excess IAA was neutralized with additional DTT, followed by incubation at 37°C for 30 minutes. Trypsin (Promega, Madison, WI) was added to the samples at a 1:25 enzyme-to-protein ratio (w/w) and incubated at 37°C for 18 hours. The reaction was stopped by adding formic acid (FA) to a final concentration of 0.5%. Samples were centrifuged at 14,800 rpm for 10 minutes. The supernatant containing digested peptides was dried and resuspended in a solution of 2% acetonitrile (ACN) and 0.1% FA for liquid chromatography-mass spectrometry/mass spectrometry (LC-MS/MS) analysis.

### LC-MS/MS analysis

Aliquots of 1 μg from the tryptic digested samples were analyzed using Liquid Chromatography-Electrospray Ionization-Tandem Mass Spectrometry (LC-ESI-MS/MS). Data acquisition for the LC-MS/MS was conducted using a Dionex Ultimate 3000 nano-LC system (Thermo Scientific, San Jose, CA) connected to a Linear Trap Quadrupole (LTQ) Orbitrap Velos mass spectrometer (Thermo Scientific, San Jose, CA) with a nano-ESI source. The injected peptides were initially purified on-line at a flow rate of 3 μL/min using a C18 Acclaim PepMap 100 trap column (75 μm I.D. × 2 cm, 3 μm particle size, 100 Å pore size, Thermo Scientific, San Jose, CA). The separation of peptides occurred on a C18 Acclaim PepMap RSLC column (75 μm I.D. × 15 cm, 2 μm particle size, 100 Å pore size, Thermo Scientific, San Jose, CA), with the column temperature maintained at 29.5°C and a flow rate of 350 nL/min during the separation process. The mobile phase consisted of solution A (97.9% water, 2% ACN, 0.1% FA) and solution B (99.9% ACN, 0.1% FA). Peptide separation was achieved using the following gradient for solution B: 5% over 10 minutes, 5%-20% over 55 minutes, 20–30% over 25 minutes, 30–50% over 20 minutes, 50%-80% over 1 minute, 80% over 4 minutes, 80%-5% over 1 minute, and 5% over 4 minutes. Data-dependent acquisition mode with two scanning events was utilized for MS/MS analysis. The first scan involved a full MS scan ranging from 400 to 2000 m/z at a mass resolution of 15,000. In the second scan event, the 10 most intense ions detected in the first scan were selected for Collision-Induced Dissociation (CID) MS/MS with an isolation width of 3.0 m/z. The normalized collision energy (CE) was set to 35%, and the activation Q value was set at 0.250. Dynamic exclusion parameters included a repeat count of 2, a repeat duration of 30 seconds, an exclusion list size of 200, and an exclusion duration of 90 seconds.

### Label-free quantitative proteomics analysis

#### Protein identification and label-free quantification (LFQ)

Raw data files from the LC-ESI-MS/MS analysis were processed using the MaxQuant software suite (version 1.6.17.0). The MS/MS spectra were searched against the *Mus musculus* reference proteome database (UniProtKB/Swiss-Prot, UP000000589, downloaded March 2023) supplemented with a reversed-sequence decoy database to empirically control the false discovery rate (FDR). The integrated Andromeda search engine was used for peptide and protein identification. Search parameters were configured as follows: Trypsin/P was specified as the proteolytic enzyme, allowing for up to two missed cleavage sites. Carbamidomethylation of cysteine was set as a fixed modification, while oxidation of methionine and acetylation of protein N-termini were specified as variable modifications. Label-free quantification was performed using the MaxLFQ algorithm ^1^, with a minimum LFQ ratio count of 2. To enhance quantification across samples, the “Match between runs” feature was enabled with a match time window of 0.7 minutes and an alignment time window of 20 minutes. The FDR for both peptide-spectrum matches (PSMs) and protein identifications was strictly controlled at 1% (*p* < 0.01).

#### Bioinformatic and statistical analysis

Subsequent data processing, statistical analysis, and visualization were performed using a combination of Perseus software (version 1.6.15.0), the R programming environment (version 4.3.1) with packages from the Bioconductor project, and GraphPad Prism (version 10.1.1). The proteinGroups.txt file generated by MaxQuant served as the input for the analysis. Proteins identified as “Potential contaminant,” “Reverse,” or “Only identified by site” were excluded from the dataset. The LFQ intensity values were then log_2_-transformed to approximate a normal distribution, a prerequisite for robust statistical testing.

To ensure high-quality quantification, proteins were filtered to retain only those quantified in at least 70% of the replicates within at least one of the three experimental groups (Control, Center, or Penumbra). The remaining missing values, which are presumed to result from signals below the instrument’s detection limit (i.e., left-censored data), were imputed. Imputation was performed by drawing random numbers from a normal distribution that was downshifted by 1.8 standard deviations and had a width of 0.3 standard deviations, relative to the measured intensity distribution of each sample.

Quality control and exploratory data analysis were conducted to assess data integrity and global trends. Raincloud plots were generated to visualize the distribution of log_2_-transformed LFQ intensities for each sample, confirming consistent data distributions post-normalization (**Supplementary Fig. 2A**). To investigate the overall proteomic variance and relationships between samples, principal component analysis (PCA) was performed on the complete, imputed data matrix using singular value decomposition on the covariance matrix (**Supplementary Figs. 2B and 2C**).

Processed protein data were analyzed using Perseus (v1.6.15.0) and the R statistical environment (v4.3.1) with Bioconductor packages. Data quality was assessed by visualizing the distribution of log_2_ intensities (e.g., raincloud plots) and by unsupervised multivariate analysis. Principal component analysis confirmed clustering of samples by treatment condition, indicating distinct proteomic profiles for Center vs. Control, etc. (as illustrated in Supplementary Fig. 2). For the volcano plot, the x-axis represents the log2 fold change, indicating the magnitude of change (fold change) in expression of quantified proteins between the two groups. Points to the right (Positive values on the x-axis) and left of the plot (negative values on the x-axis) with a fold change (FC) above threshold lines of Log_2_FC at ± 1 (0.5 ≥ fold change ≥ 2) respectively denote down- and up-regulation of quantified proteins in either center or penumbra groups compared to the center group. The y-axis represents the negative log2-transformed *p*-values (*p*), which are used to assess the statistical significance of the differential expression. Points in the upper part of the volcano plot, above the threshold line of −Log2(p) ≥ 4.35, are considered statistically significant. Larger values on the y-axis suggest greater statistical significance. Differential protein expression between 2 groups (Center vs. Control and Penumbra vs. Control) was assessed using an unpaired two-tailed t-test. Proteins with adjusted *p* < 0.05 and 0.5 ≥ fold change ≥ 2 were considered differentially expressed (Fig. 5). The lists of differentially expressed proteins (DEPs) for each comparison were further analyzed for overlaps: Venn diagrams and UpSet plots were generated to show shared and unique DEPs between the Center vs. Control and Penumbra vs. Control groups (Fig. 8A–D). DEPs were defined as proteins with an adjusted *p* < 0.05 and an absolute log_2_ fold change > 0.585 (≥ 1.5-fold). To visualize expression patterns, DEPs were subjected to unsupervised hierarchical clustering (Euclidean distance, complete linkage) and displayed in a heatmap (Fig. 8E), highlighting distinct protein expression signatures in the different exposure regions.

All quantitative data are presented as mean ± SEM. Group comparisons for the cell-based assays (MTT, LDH, and densitometry of Western blots) were conducted by one-way ANOVA followed by Tukey’s post hoc test using GraphPad Prism software (v10.1.1, GraphPad Software, Boston, MA, USA). *p* < 0.05 was considered statistically significant for all tests. In figures, significance levels are indicated by asterisks: *p* < 0.05 (*), *p* < 0.01 (**), *p* < 0.001 (***), and *p* < 0.0001 (****). For the proteomics data, statistical criteria for differential expression were applied as described above (moderated t-tests with false discovery rate [FDR] correction).

#### Functional enrichment and pathway analysis

To gain insight into the biological pathways and processes perturbed by mmWave exposure, identified DEPs were subjected to gene ontology and pathway enrichment analysis. Over-representation analysis was performed using the g:Profiler tool (version e109_eg56_p17) for GO terms in the categories of Biological Process (BP), Molecular Function (MF), and Cellular Component, as well as KEGG and Reactome pathway databases. Enrichment was tested separately for up-regulated and down-regulated protein lists from each comparison. GO terms or pathways with a Benjamini-Hochberg FDR-adjusted *p* < 0.10 were considered significantly enriched. The enrichment results were summarized and visualized in a heatmap format, indicating the normalized enrichment scores of selected GO terms across all samples. This allowed for the visualization of how specific functional categories were differentially affected in the Center, Penumbra, and Control conditions (Fig. 9A–C). To identify protein associations and assess the enrichment of disease-related and functional pathways within the dataset, biological pathway and network analysis was performed using Qiagen’s Ingenuity Pathway Analysis (IPA) software. Predicted activation or inhibition of specific functions was inferred based on z-scores, with positive values indicating activation and negative values indicating inhibition (Fig. 9D). In addition, to investigate the role of key biological features, we used PathwayStudio v10.0 (Elsevier, UK) to analyze protein-protein interactions, molecular functions, and associated biological processes (Fig. 10).

#### Ethical compliance

This study did not involve any human participants or live vertebrate animals. The use of the N2a mouse neuroblastoma cell line (ATCC CCL-131) does not require specific ethical approval, as it is a commercially available cell line. All experimental protocols, including cell handling, directed energy exposure, and biohazard safety procedures, were conducted according to institutional biosafety guidelines and standards for cell culture research. Since only established cell lines were used, no additional ethical clearance was necessary for these in vitro experiments.

## Results

### mmWave DE exposure induces a spatially-graded decline in cell viability and integrity

To investigate the cytotoxic effects of mmWave DE, N2A neuroblastoma cells were exposed to 34.14 GHz irradiation for 48 hours using an experimental setup designed to create a gradient of exposure intensity (Fig. 1). We first assessed the impact of this exposure on cellular morphology, viability, and membrane integrity, establishing a clear relationship between proximity to the irradiation source and the severity of cellular damage.

**Figure 2:**
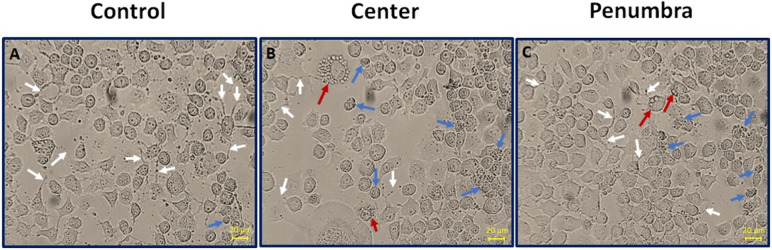
Effects of DE exposure on the morphology of N2A cells. (A) Control, (B) Center, and (C) Penumbra groups. Cell photographs were obtained using an inverted phase-contrast light microscope at 48 hours after the last exposure. Scale bar is 20 μm (Magnification 250×). White arrow: cell processes, Blue arrows: rounded (degenerative) cells, Red Arrows: cells with vacuoles (vacuolization).

The mmWave DE irradiation can induce various morphological changes in cells, which may include alterations in cell morphology. These changes are indicators of cellular stress, damage, or adaptation to the mmWave DE exposure and can manifest in several ways. When cells are unhealthy and begin to die, their shape, size, and structure change. The cell bodies round up, processes are degraded, vacuoles form, and a significant amount of debris is generated. In our study, the cells of the central group directly exposed to mmWave DE irradiation exhibited more pronounced morphological changes than the cells in the other groups. According to Fig. 2, one of the characteristics of healthy neuroblastoma cells, the processes, were degraded and severely reduced in the cells under direct radiation (center group) compared to the cells in the control and penumbra groups. Additionally, the center group exhibited more cells that had changed shape, size, and structure, becoming smaller and more rounded than the other groups. Another characteristic of damaged cells is the formation of vacuoles. According to our findings, the number of vacuoles formed among the cells under direct radiation (center group) was more than in the other groups (control and penumbra).

**Figure 3:**
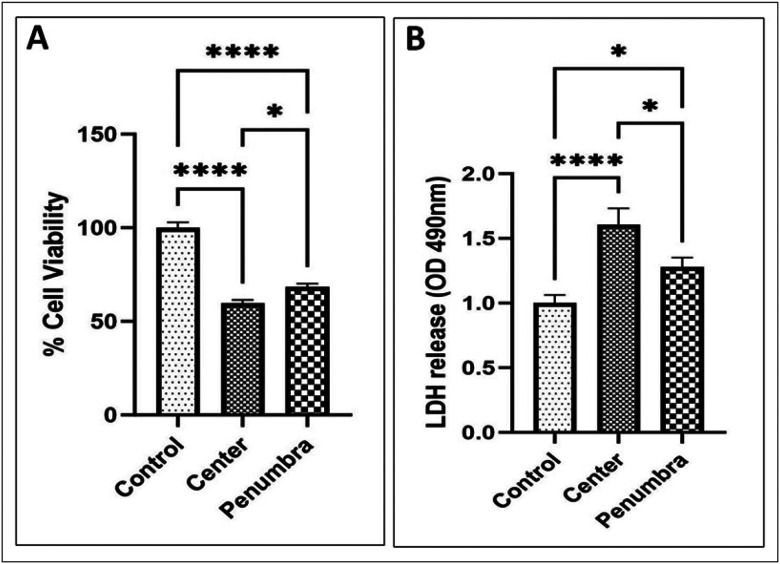
Effects of DE exposure on viability, cell membrane integrity, and cell death of N2A cells. (A) The MTT assay was used to measure the effect of DE exposure on the viability of N2A cells 48 hours after the last exposure. (B) The LDH leakage assay was employed to quantify LDH release, which was used as an indicator of cell membrane integrity and cytotoxicity in N2A cells 48 hours after the last DE exposure. The results are presented as the mean ± SEM. Asterisks indicate statistical significance between groups (*p < 0.05, ****p < 0.0001). MTT, [3-(4,5-Dimethylthiazol-2-yl)-2,5-Diphenyltetrazolium Bromide]; LDH, lactate dehydrogenase.

Cell viability was assessed 48 h after exposure to the last mmWave DE irradiation using the MTT assay. The results were expressed as the percentage of cell viability relative to control cells. A significant loss of viability was observed in mmWave-exposed cells, especially in the Center zone (Fig. 3A). Forty-eight hours after exposure, MTT assay results showed that the viable cell percentage in the Center group dropped sharply relative to the Control group. One-way ANOVA analysis confirmed an overall exposure effect (****p < 0.0001), and post-hoc tests revealed that Center viability was only ~ 50% of control (****p < 0.0001), indicating a pronounced cytotoxic effect. The Penumbra group also exhibited a viability reduction (to roughly ~ 80% of control, ****p < 0.0001), although this decrease was significantly milder than in the Center (Center vs. Penumbra *p < 0.05). These results demonstrate a spatial gradient of cytotoxicity, with cell survival inversely proportional to proximity to the mmWave source.

### Cell viability and LDH release assays

The cytotoxic effects of mmWave DE irradiation on N2A cells were quantitatively assessed by measuring LDH release 48 h after the last exposure. This assay evaluates the integrity of the cell membrane by measuring LDH enzyme leakage into the culture medium, indicative of cellular damage and cell death. The results were presented as the cell death ratio relative to the control. As shown in Fig. 3B, LDH release, measured as a percentage of total cellular LDH, was minimal in Control cells but markedly increased in the Center group, reflecting extensive membrane damage. Center-exposed cells released roughly 3–4-fold more LDH than controls (****p < 0.0001), indicative of significant cell lysis. Cells in the penumbra group also exhibited an increased LDH release compared to control (*p < 0.05), but the increase was less pronounced than in the center group. Together, the viability and LDH assays confirm that mmWave DE exposure induces dose-dependent cell death, with the highest lethality and membrane compromise occurring at the beam center.

### mmWave DE exposure triggers proteolytic degradation of key cytoskeletal proteins

**Figure 4:**
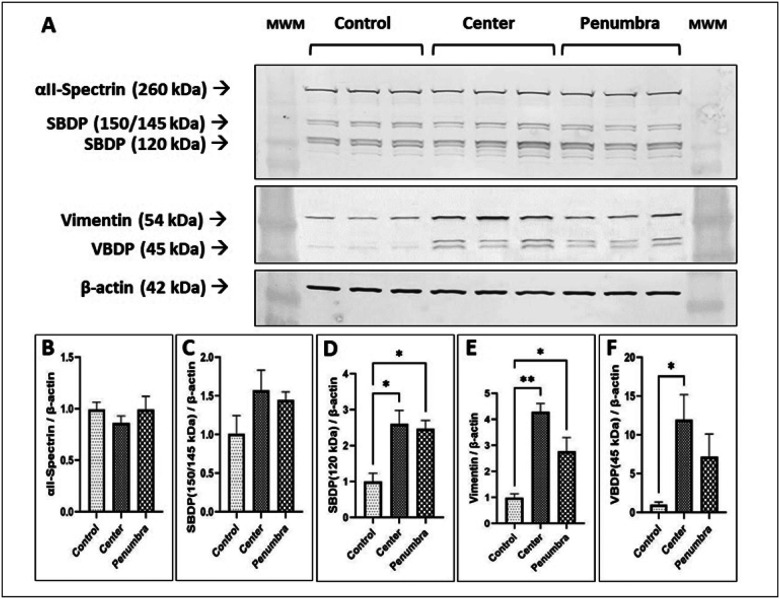
Western blot analysis of the expression of proteins associated with cell membrane integrity. (A) Western blot analysis showing differential expression of αII-Spectrin, Vimentin, their breakdown products, and β-actin used as a western blot loading control across three groups: control, center, and penumbra in DE-exposed N2A cells. Densitometric analysis of the bands reveals the relative levels of (B) αII-Spectrin, (C, D) SBDP (150/145 and 120 kDa), (E) Vimentin, and (F) VBDP (45 kDa), all normalized to β-actin (42 kDa). The results are presented as the mean ± SEM. Asterisks indicate statistical significance compared to the control group (*p < 0.05, **p< 0.01). SBDP, αII-Spectrin Breakdown Product; VBDP, Vimentin Breakdown Product. MWM – Molecular weight markers

To evaluate cytoskeletal protein damage, we examined αII-Spectrin and Vimentin levels, as well as their proteolytic fragments, by Western blot (Fig. 4A). These markers of structural integrity exhibited exposure-dependent proteolysis patterns consistent with cellular stress. This analysis was conducted to determine the impact of DE exposure on cellular structures, particularly in different exposure zones labeled as center and penumbra. Western blot analysis revealed noticeable differences in the breakdown of αII-Spectrin and Vimentin between the control, center, and penumbra groups (Fig. 4A).

Full-length αII-Spectrin (~ 260 kDa) was abundantly expressed in all groups with no significant change in band intensity between Control, Penumbra, and Center samples. (Fig. 4B). However, cleaved Spectrin fragments were differentially affected. The ~ 150 kDa Spectrin breakdown product (SBDP150, often generated by calpain) showed no appreciable difference among groups (Fig. 4C). In contrast, the ~ 120 kDa fragment (SBDP120, a hallmark caspase/calpain product) was significantly elevated in both Center and Penumbra exposures relative to Control (both *p* < 0.05; Fig. 4D). Densitometry confirmed ~ 1.5–2-fold increases in SBDP120 in the exposed groups, indicating enhanced Spectrin proteolysis in mmWave-treated cells (most pronounced in the Center zone). (Fig. 4D).

The intermediate filament protein Vimentin (~ 54 kDa) displayed a distinct expression shift upon exposure. Surprisingly, the Center group showed an increase in full-length Vimentin levels to ~ 140% of control (*p* < 0.01), and the Penumbra group to ~ 120% (*p* < 0.05), suggesting an exposure-induced accumulation or upregulation of Vimentin (Fig. 4E). Concomitantly, a Vimentin breakdown product (~ 45 kDa, VBDP) emerged. This VBDP was markedly elevated in Center samples (*p* < 0.05 vs. Control), whereas Penumbra showed only a slight, non-significant increase (Fig. 4F). The enrichment of VBDP in Center-exposed cells signifies active Vimentin cleavage under high-intensity exposure. Collectively, the Western blot results demonstrate that mmWave DE exposure triggers cytoskeletal protein degradation, with robust Spectrin cleavage and intermediate filament disruption correlating with exposure intensity. These molecular signs of protein breakdown align with the observed loss of structural integrity in damaged cells.

### Label-free quantitative proteomics reveals exposure-dependent protein alterations

To gain an unbiased, systems-level view of the cellular response, we performed label-free quantitative proteomics on N2A cells from Control, Penumbra, and Center groups. This in-depth analysis (using n = 6 control, 8 penumbra, 4 center biological replicates) identified > 2,500 proteins across all samples, providing a comprehensive proteomic dataset. The results show that mmWave DE exposure induces broad proteome remodeling, with distinct signatures in the Center and Penumbra zones.

### Global proteome analysis confirms high data quality and exposure-dependent stratification

Initial quality control assessments confirmed the high quality and consistency of the proteomic data. Raincloud plots of the log_2_-transformed LFQ intensities showed highly similar distributions across all biological replicates, indicating successful normalization and the absence of significant technical bias (**Supplementary Fig. 2A**).

To visualize the overall impact of mmWave DE exposure on the N2A cell proteome, we employed PCA. The resulting scores plot demonstrated a striking and robust separation of the three experimental groups (**Supplementary Fig. 2B**). The Control samples formed a tight, distinct cluster, indicative of a stable baseline proteome. The Center and Penumbra groups also formed their discrete clusters, which were separated from the Control group and each other. This separation demonstrates that direct (Center) and indirect (Penumbra) exposure induced reproducible and distinct proteomic shifts. The primary axis of variation, Principal Component 1 (PC1), accounted for 21.3% of the total variance and maximally separated the Control group from the exposed groups. Notably, the Center group exhibited the most significant displacement from the Control cluster along PC1, while the Penumbra group occupied an intermediate position. This spatial arrangement provides compelling evidence for a proteome-wide response gradient, where the magnitude of the cellular perturbation is directly proportional to the proximity to the mmWave DE source. A three-dimensional PCA plot further reinforced this clear separation in multivariate space (**Supplementary Fig. 2C**).

### Direct mmWave exposure induces profound proteomic remodeling in the center zone

**Figure 5:**
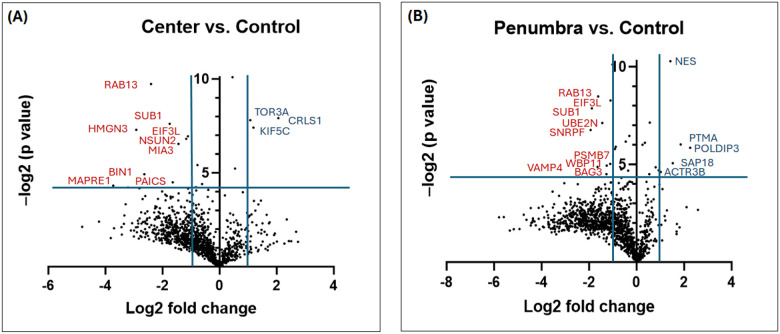
Differential Protein Expression Profiles in Response to mmWave Directed Energy Exposure. Volcano plots illustrating global differential protein expression between exposed and unexposed N2A cells, generated from label-free quantitative proteomics analysis. (A) Center vs. Control comparison: cells in the central region of mmWave DE exposure (n = 4) relative to control cells (n = 6), with significantly upregulated (blue, right quadrant) and downregulated (red, left quadrant) proteins based on thresholds of |log_2_ fold change| ≥2 with *p* < 0.05. (B) Penumbra vs. Control comparison: peripheral DE-exposed cells (n = 8) exhibiting upregulated and downregulated proteins compared to control. Only proteins meeting both fold-change and significance criteria are labeled by their UniProt accession numbers.

Differential Expression in Center vs Control: Quantitative comparison of the Center group to Control revealed extensive proteomic changes. We identified 181 proteins as significantly dysregulated in Center-exposed cells (adjusted *p* < 0.05, fold-change > 1.5), comprising 30 upregulated and 151 downregulated proteins (Fig. 8A–D). The volcano plot (Fig. 5A) illustrates this broad shift, with numerous proteins exhibiting high-magnitude decreases in abundance in the Center.

**Figure 6:**
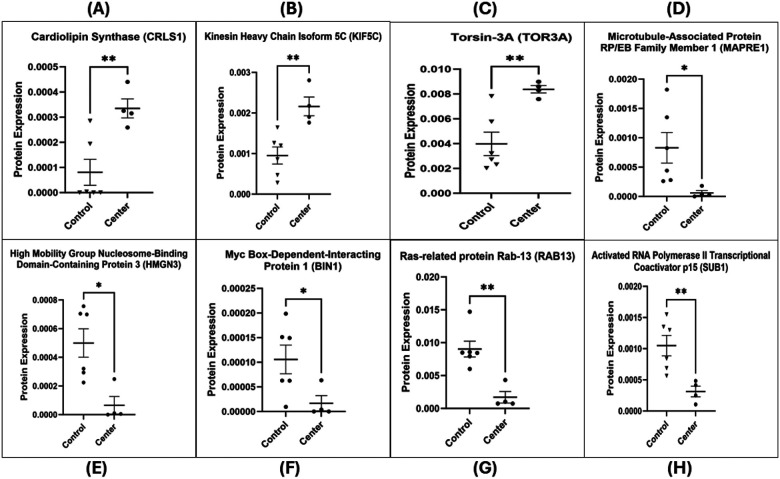
Top differentially expressed proteins in Center-exposed cells compared to Control. Bar graphs depict the normalized protein expression levels (mean ± SEM) of key upregulated and downregulated hits identified from the LC-MS/MS-based label-free proteomics analysis comparing mmWave DE-exposed Center wells (*n* = 4) to Control wells (*n* = 6). (A–C) Significantly upregulated proteins in the Center group include Cardiolipin Synthase (CRLS1), Kinesin Heavy Chain Isoform 5C (KIF5C), and Torsin-3A (TOR3A), suggesting mitochondrial and cytoskeletal adaptations. (D–H) Downregulated proteins include Microtubule-Associated Protein RP/EB Family Member 1 (MAPRE1), High Mobility Group Nucleosome-Binding Protein 3 (HMGN3), Myc Box-Dependent-Interacting Protein 1 (BIN1), Ras-related Protein Rab-13 (RAB13), and Transcriptional Coactivator p15 (SUB1), indicating structural, transcriptional, and vesicular trafficking disruptions. Statistical comparisons were performed using an unpaired two-tailed Student’s *t*-test. Asterisks denote significance: *p < 0.05, **p < 0.01. Protein IDs are shown in parentheses (UniProt accession numbers).

Consistent with a severe stress response, many core cellular proteins were strongly downregulated in the Center zone. For example, MAPRE1 (EB1, a microtubule-associated protein) was among the most suppressed (log_2_fold-change ≈ − 3.7), alongside other key regulators such as HMGN3 (chromatin binding, − 2.9), BIN1 (membrane curvature/scaffold protein, − 2.6), RAB13 (vesicle trafficking GTPase, − 2.4), and SUB1 (transcriptional coactivator, − 1.8). These pronounced losses suggest a collapse in cytoskeletal organization, gene regulation, and transport processes under direct exposure. Conversely, a smaller set of proteins was upregulated in the Center zone, including stress or metabolism-related proteins. Notable examples are CRLS1 (cardiolipin synthase, log_2_ + 2.06), KIF5C (kinesin family motor protein, + 1.18), and TOR3A (Torsin-3A, + 1.07) which increased in abundance. The complete list of DEPs is provided in **Supplementary Table 1**, and representative top hits are highlighted in Fig. 6. Overall, direct mmWave exposure in the Center elicited a massive proteomic response characterized by widespread protein downregulation with selective upregulation of specific stress-associated proteins.

### The penumbra zone exhibits a milder but significant proteomic signature

**Figure 7:**
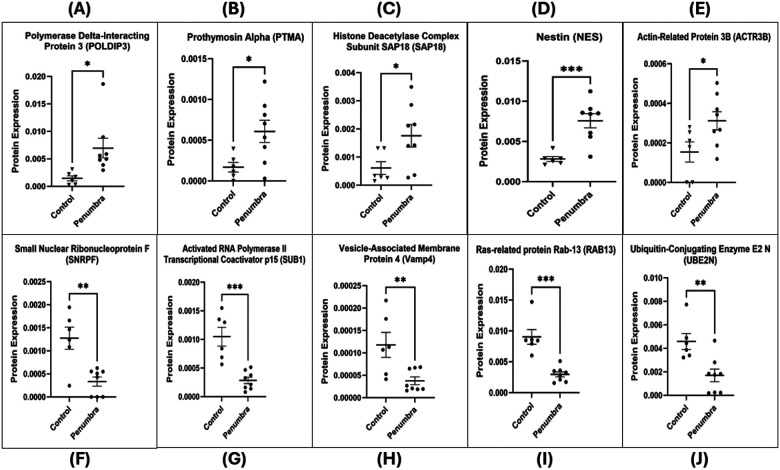
Top differentially expressed proteins in Penumbra-exposed cells compared to Control. Normalized protein expression levels (mean ± SEM) are shown for selected upregulated and downregulated proteins identified from LC-MS/MS-based label-free proteomics analysis comparing Penumbra-exposed wells (*n* = 8) to Control wells (*n* = 6). (A–E) Proteins significantly upregulated in the Penumbra group include Polymerase Delta-Interacting Protein 3 (POLDIP3), Prothymosin Alpha (PTMA), Histone Deacetylase Complex Subunit SAP18 (SAP18), Nestin (NES), and Actin-Related Protein 3B (ACTR3B), indicating transcriptional activity, chromatin remodeling, and cytoskeletal adaptation. (F–J) Downregulated proteins include Small Nuclear Ribonucleoprotein F (SNRPF), RNA Polymerase II Transcriptional Coactivator p15 (SUB1), Vesicle-Associated Membrane Protein 4 (VAMP4), Ras-related Protein Rab-13 (RAB13), and Ubiquitin-Conjugating Enzyme E2 N (UBE2N), suggesting impaired RNA processing, vesicular trafficking, and protein turnover. Statistical comparisons were performed using unpaired two-tailed Student’s *t*-test. Significance is indicated as follows: *p < 0.05, **p < *0.01*, ***p < *0.001*. Protein IDs are shown in parentheses (UniProt accession numbers).

Differential Expression in Penumbra vs. Control: The Penumbra exposure, although milder, still produced a significant proteomic signature distinct from that of controls. In total, 34 proteins (10 up, 24 down) were significantly altered in Penumbra-exposed cells relative to Control (adjusted *p* < 0.05; Fig. 5B). This represents a smaller subset of changes (roughly one-fifth the number seen in the Center), consistent with the less intense exposure. The pattern of changes in Penumbra cells suggests a sublethal stress response. Several proteins involved in RNA processing and stress responses were upregulated – for instance, POLDIP3 (polymerase delta-interacting protein 3, log_2 + 1.86), PTMA (prothymosin-α, + 1.86), and SAP18 (histone deacetylation, + 1.52) were among the top increases. In addition, markers of neural or developmental state, such as nestin (an intermediate filament protein, + 1.43) and actin cytoskeletal regulators such as ACTR3B (Actin-related protein 3B, + 1.02) increased in Penumbra cells, possibly indicating cytoskeletal reorganization. On the other hand, 24 proteins were downregulated in the Penumbra (though generally with more minor magnitude changes than in the Center). These included SNRPF (small nuclear ribonucleoprotein F, log_2_ − 1.93) and SUB1 (− 1.88), which implicated impacts on mRNA splicing/transcriptional processes, as well as VAMP4 (vesicle-membrane protein, − 1.64) and RAB13 (− 1.61), indicating dampened vesicle transport and membrane trafficking. Meanwhile, UBE2N (ubiquitin-conjugating enzyme E2 N, − 1.44) was also reduced, which may weaken the cell’s ability to repair DNA and respond to stress. The significant DEPs in Penumbra are summarized in Fig. 7. While more limited in scope, the Penumbra proteome changes confirm that even peripheral mmWave exposure induces measurable stress-related protein alterations, albeit far fewer than direct exposure.

### Comparative analysis defines unique and overlapping proteomic responses to the exposure gradient

**Figure 8:**
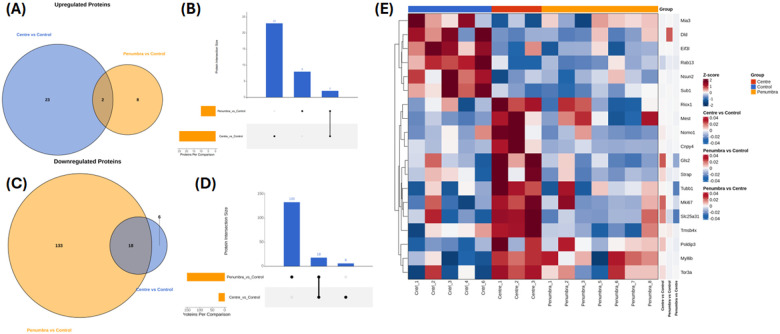
Differential proteomic profiling of mmWave DE-exposed N2A cells from center and penumbra regions. (A, C) Venn diagrams showing the overlap of significantly upregulated (A) and downregulated (C) proteins in the Center vs. Control and Penumbra vs. Control groups. (B, D) UpSet plots illustrating the number of DEPs and their intersections between the Center and Penumbra groups compared to Control, for upregulated (B) and downregulated (D) proteins, respectively. Bars indicate set size; connected dots represent shared or unique DEPs between comparisons. (E) Hierarchical clustering heatmap of DEPs across all samples, based on Z-score normalized log_2_-transformed LFQ intensities. Each column represents a biological replicate (n=3–4 per group), and each row corresponds to a DEP. Heatmap clustering was performed using Euclidean distance and complete linkage. Statistically significant pairwise comparisons are annotated on the right of each protein, based on adjusted *p*-values (FDR < 0.05) computed using moderated t-tests (limma). Missing values were imputed with a left-censored normal distribution (width = 0.3, downshift = 1.8). DEPs were defined as proteins with an adjusted *p* < 0.05 and an absolute log_2_ fold change > 0.585 (≥1.5-fold).

To distinguish between the core cellular response to mmWave DE and the effects of exposure intensity, we compared the DEP lists from both conditions. Venn diagrams and UpSet plots revealed that a core set of 8 upregulated and 18 downregulated proteins was shared between the Center and Penumbra groups, representing a common signature of mmWave DE-induced stress (Fig. 8A–D). However, most DEPs were unique to the Center condition, which exhibited 22 unique upregulated and 133 unique downregulated proteins. This vastly outnumbered the distinctive changes in the Penumbra group (2 upregulated, 6 downregulated), underscoring the severity of the direct exposure.

Unsupervised clustering of the combined DEP dataset (all 201 proteins that were significant in either comparison) further illustrated the divergence between groups. The heatmap (Fig. 8E) cleanly clustered samples by exposure condition, segregating all Control, Penumbra, and Center samples into separate branches. Control samples clustered together with a distinct protein expression profile, while Penumbra and Center samples clustered apart, each defined by their characteristic pattern of protein changes. The heatmap also revealed distinct co-regulated protein clusters associated with each condition. For example, a cluster of proteins related to cell proliferation and cytoskeletal organization (including Mki67/Ki-67, a proliferation marker; Tubb1, a tubulin; and Strap, a signaling scaffold) was strongly downregulated exclusively in Center samples. This exclusive downregulation in Center cells aligns with the severe growth impairment and structural breakdown observed under high exposure. Meanwhile, a different subset of proteins (e.g., Tor3a, Mybbp1a) showed coordinated changes in both Center and Penumbra groups, reflecting the common stress response signature. Thus, the clustering analysis confirms that Center and Penumbra exposures elicit both shared and unique proteomic changes, with the magnitude and breadth of changes greatest in the Center group.

### Systems-level analysis links proteomic changes to perturbations in cellular structure, protein homeostasis, and metabolism

**Figure 9:**
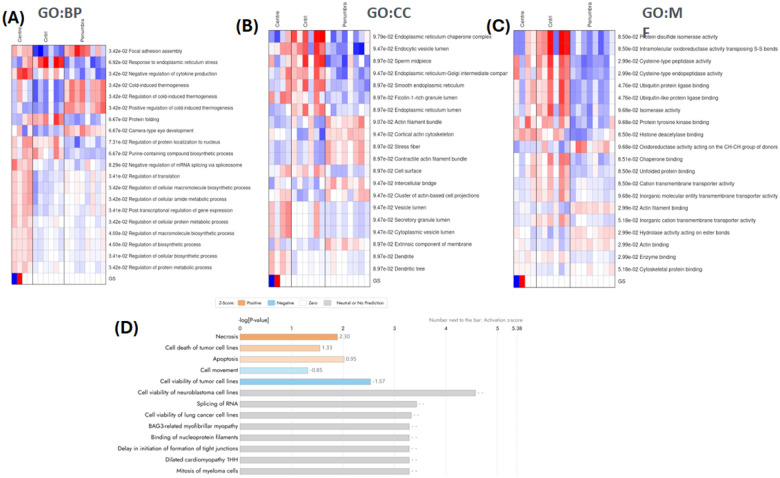
Systems-level Functional and Pathway Enrichment Analysis of mmWave DE-Exposed Neuroblastoma Cells. (A–C) Heatmaps of enriched Gene Ontology terms for Biological Process (GO: BP), Cellular Component (GO: CC), and Molecular Function (GO: MF) categories, derived from label-free quantitative proteomics in the Control, Penumbra, and Center groups following mmWave DE exposure. Functional enrichment was performed using over-representation analysis (ORA) on DEPs; adjusted *p* < 0.05, |log_2_FC|>2). Color intensity reflects normalized Z-scores representing the direction and magnitude of enrichment per sample: red/orange indicates positive enrichment (activation), blue indicates negative enrichment (suppression), and white indicates no prediction or neutral score. Each column represents an individual biological replicate, and each row corresponds to an enriched GO term, annotated with its adjusted *p*-value. (A) GO: BP. (B) GO: CC. (C) GO: MF. (D) IPA of the Penumbra vs. Control comparison identifies significantly enriched cellular functions and disease-associated pathways. Each bar represents a predicted function, ranked by statistical significance (−log₁₀[*p*-value], x-axis). Bar color indicates predicted activation state: orange for activation (positive Z-score), blue for inhibition (negative Z-score), and gray for functions without a directional prediction. Activation Z-scores are displayed at the end of each bar. Top enriched functions include activation of necrosis, tumor cell death, and apoptosis, alongside predicted inhibition of cell movement and cell viability in tumor and neuroblastoma cells, reflecting sublethal but stress-associated effects in the Penumbra region.

**Figure 10:**
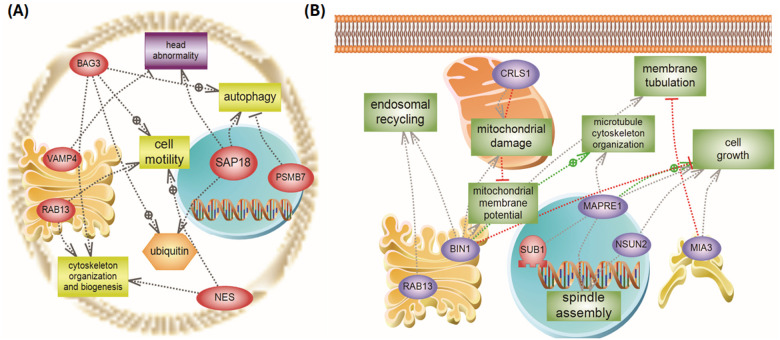
Protein Interaction and Functional Association Maps of DE-Regulated Proteins in Penumbra and Center Zones Following mmWave Exposure. (A) Network map of Penumbra vs. Control comparison, highlighting protein–process associations identified using *Pathway Studio v10.0*. Downregulated and upregulated proteins (red ovals) are integrated into functional cellular pathways (colored boxes). Relationships between proteins and biological processes such as autophagy, cell motility, cytoskeleton organization, and ubiquitin-mediated regulation are shown. Nodes such as SAP18, NES, VAMP4, RAB13, PSMB7, and BAG3 are central to modulating proteostasis and sublethal stress responses. Dashed arrows indicate known functional interactions curated from literature-based databases. (B) Network map of Center vs. Control comparison showing enriched mechanistic pathways associated with severe mmWave-induced injury. Highlighted DEPs (purple ovals), including CRLS1, MAPRE1, BIN1, SUB1, MIA3, and NSUN2, are mapped to processes such as mitochondrial damage, microtubule cytoskeleton organization, spindle assembly, membrane tubulation, and cell growth suppression. Red edges indicate inhibition, green edges indicate activation, and grey dashed lines represent known associative links. These maps were generated using Pathway Studio (Elsevier) to visually integrate DEPs; adjusted *p*< 0.05, |log_2_FC| > 2) with curated cellular processes and pathways.

To further elucidate the cellular response to mmWave DE exposure, we performed systems-level analyses of DEPs using GO enrichment and pathway-centric network mapping. These analyses provide insight into how mmWave-induced proteome alterations converge on biological functions, cellular structures, and disease-relevant signaling programs in a spatially dependent manner. The results of the GO enrichment analysis are summarized in Fig. 9, while functional interaction networks generated using Pathway Studio are presented in Fig. 10.

GO-based enrichment analysis (Fig. 9A–C) identified distinct stress-response signatures in the Center and Penumbra groups. In the BP category (Fig. 9A), the Center group exhibited strong enrichment in terms related to proteotoxic stress, such as “response to endoplasmic reticulum stress,” “protein folding,” and “regulation of protein catabolic process,” indicating disruptions in protein homeostasis. At the same time, essential processes for gene regulation and biosynthesis, including “post-transcriptional regulation of gene expression” and “mRNA splicing, via spliceosome,” were significantly downregulated, suggesting an energy-saving shift in transcriptional and translational activities.

In contrast, the Penumbra group exhibited moderate enrichment of stress-related processes, including terms related to protein repair and cytoskeletal maintenance (e.g., “response to stress” and “protein stabilization”). Still, it lacked the full collapse of gene expression pathways observed in the Center zone. These patterns indicate a graded activation of proteostasis mechanisms, where the Penumbra initiates adaptive responses, while the Center undergoes a catastrophic loss of regulatory and synthetic capacity.

In the Cellular Component (CC) domain (Fig. 9B), Center-exposed cells showed downregulation of structural elements such as the “actin cytoskeleton,” “intermediate filaments,” and “focal adhesion,” supporting the extensive cytoskeletal degradation observed in the Western blot data. Additional dysregulation of components such as the “endoplasmic reticulum lumen” and “Golgi intermediate compartment” in the Center group suggested widespread disruption of the secretory pathway. Penumbra cells also displayed modest perturbations in cytoskeletal compartments but retained partial structural coherence, in agreement with their less severe phenotypic damage.

GO terms in the MF category (Fig. 9C) further underscored the divergence between Center and Penumbra responses. Both groups showed enrichment for “chaperone binding” and “unfolded protein binding,” signaling engagement of the unfolded protein response (UPR). However, only the Center group demonstrated substantial loss of “RNA binding,” “translation initiation factor activity,” and other functions essential for mRNA metabolism and ribosomal loading—highlighting a transition from stress adaptation in the Penumbra to transcriptional shutdown and proteostasis failure in the Center.

Figure 9D presents the results of IPA performed on DEPs from the Penumbra vs. Control comparison, providing mechanistic insights into the sublethal cellular response to peripheral mmWave DE exposure. The analysis highlights a set of biological functions that are either significantly activated or inhibited based on z-score predictions and *p*-value rankings. Notably, cell death-related processes such as necrosis, tumor cell death, and apoptosis were predicted to be activated (orange bars), indicating that Penumbra-exposed cells are primed for stress-induced degeneration, even in the absence of overt viability loss. Conversely, several vital cellular functions were predicted to be inhibited, including viability of neuroblastoma cells, RNA splicing, protein translation, and cell movement (blue bars). These inhibitory trends align with GO-based observations of translational repression and cytoskeletal impairment in the Penumbra, suggesting functional compromise despite the lack of widespread protein collapse. Collectively, the IPA output underscores that peripheral mmWave exposure initiates a molecular phenotype marked by suppressed biosynthetic capacity and incipient pro-death signaling, representing a transitional state between survival and degeneration.

To complement this statistical enrichment view, we constructed biological association networks using Pathway Studio, focusing on the top DEPs in each exposure group (Fig. 10). These interaction maps contextualize the proteomic shifts within known functional pathways and disease mechanisms. In the Penumbra vs. Control network (Fig. 10A), upregulated proteins such as SAP18, POLDIP3, NES, and BAG3 mapped onto pathways involved in autophagy, chromatin remodeling, cytoskeletal integrity, and ubiquitin-mediated proteolysis. Downregulated targets, including RAB13 and VAMP4, were associated with vesicular trafficking and neurotransmitter release. Collectively, this map supports a sublethal cellular stress response, characterized by compensatory remodeling of protein degradation pathways and moderate suppression of membrane trafficking.

In the Center vs. Control network (Fig. 10B), the topology revealed a starkly different architecture, marked by the loss of structural, mitochondrial, and transcriptional components. Central nodes such as MAPRE1, BIN1, SUB1, and HMGN3 were strongly downregulated and linked to critical pathways, including microtubule polymerization, membrane curvature sensing, and RNA synthesis regulation. Upregulated effectors like CRLS1 and TOR3A were related to mitochondrial lipid metabolism and endoplasmic reticulum stress responses, respectively, highlighting attempts at intracellular repair amidst systemic collapse. Notably, red interaction lines denoted inhibitory links, whereas green arrows denoted activation, visually underscoring the oppositional shifts in molecular homeostasis between adaptation (Penumbra) and dysfunction (Center).

Taken together, our results provide complementary perspectives on the spatially graded molecular effects of mmWave DE exposure. The GO enrichment heatmaps (Fig. 9) define statistically significant pathway-level alterations in gene expression, structure, and function, while the curated networks (Fig. 10) reveal how key protein nodes integrate into biological processes that govern neuronal integrity, survival, and disease susceptibility. The convergence of pathway-level stress activation (e.g., UPR, autophagy) with cytoskeletal collapse and transcriptional silencing provides a mechanistic framework that connects mmWave DE exposure to neurodegeneration-relevant processes, warranting further in vivo and longitudinal investigation.

## Discussion

This study provides the first in-depth proteomic analysis of the cellular response to 34.14 GHz mmWave DE, revealing a sophisticated, spatially dependent neurotoxic mechanism. The observed morphological changes in N2A cells exposed to mmWave DE irradiation highlight critical cellular responses, suggesting significant stress and potential damage, similar to the findings by Li et al. (2021) on impairments in neurite outgrowth due to RF-EMR exposure (15). Such cellular alterations, characterized by changes in cell size, shape, and the formation of vacuoles, particularly in the center group, which were directly exposed to mmWave DE irradiation, indicate a severe disruption in cellular integrity and function, often preceding apoptosis or similar cell death processes. These changes are consistent with those observed by Zymantiene et al. (2020) in cortical neurons under identical conditions, suggesting a link to cellular stress responses and potential long-term neurodegenerative processes (16). In addition, the study highlighting the impact of global system for mobile communication (GSM) mobile phone EMFs on rat brains, causing significant neuronal damage, supports our findings. The observed significant neuronal damage in the cortex, hippocampus, and basal ganglia due to electromagnetic exposure parallels the morphological changes noted in our study, emphasizing the potential harmful effects of high-frequency EMR (19). This correlation underscores the need for further investigation to comprehend and mitigate the risks associated with electromagnetic exposure in technological and medical settings.

Despite efforts to minimize heating effects in mmWave DE setups, non-thermal effects such as disruptions in cellular membranes and signaling pathways still play a substantial role in cellular responses (20). According to a study conducted by Pal (2022), even low-level EMR can lead to significant biological changes (7). The increase in cellular features, such as vacuolization and rounding, in areas directly exposed to mmWave DE raises concerns about the pathophysiological impacts, particularly under conditions that mimic real-world exposure scenarios, like those in military and telecommunications applications. This could potentially link to syndromes such as Havana syndrome, as recent studies suggest a connection between such conditions and pulsed RF energy exposure, highlighting the need for further research to understand and mitigate emerging technological risks (10, 13, 18). Additional studies by Foster and Vijayalaxmi (2021) and the National Academies of Sciences, Engineering, and Medicine (2020) emphasize the urgent need for more reliable bioeffects studies on high-band 5G frequencies and their health implications (14, 17).

Given the significant morphological changes observed in N2A cells, which are indicative of cellular stress and potential damage, it becomes imperative to further investigate the effects of mmWave DE exposure on more definitive cellular endpoints such as viability, cell membrane integrity, and cell death. Understanding these aspects will provide a clearer picture of the cellular dynamics post-exposure, particularly regarding cytotoxicity and the potential for irreversible cellular dysfunction. In this regard, the effects of DE exposure on viability, cell membrane integrity and cell death were studied.

The significant reduction in cell viability observed in N2A cells after exposure to mmWave DE, as evidenced by the MTT assay, underscores the cytotoxic potential of mmWave DE irradiation, which has been previously demonstrated in similar studies. These findings underscore the potential cytotoxic impact of mmWave irradiation under the conditions tested, with more pronounced effects closer to the irradiation source (20, 21). This reduction in viability was more marked in the center group, located directly under the DE source, than in the penumbra group, indicating a dose-dependent cytotoxic effect. Such findings align with prior studies demonstrating the harmful effects of RF-EMR on cellular health and functionality, as noted by Li et al. (2021), where similar exposures led to significant cellular impairment in neuron-like cells (15). The gradient effect observed in viability between the center and penumbra groups highlights the importance of spatial exposure metrics in assessing the risk associated with mmWave technologies. This aspect has been discussed in the broader context of RF safety assessments by Foster and Vijayalaxmi (2021)(17).

Parallel assessments of cell membrane integrity via LDH release assays further validated the disruptive impact of mmWave DE on cellular structures. LDH is a soluble enzyme found in the cytoplasm of nearly all cell types and is released into the surrounding environment when the cell membrane is compromised. Consequently, LDH release is a marker of membrane damage and a hallmark of cell death, providing a means to measure the proportion of living and dead cells. This release is typically associated with necrosis, a form of cell death where cells swell and their internal structures rupture, leading to the disintegration of the cell membrane (22). EMF exposure can have significant effects on cell membrane integrity and cell death, depending on the frequency, intensity, and duration of exposure (23). The results from this study contribute to a growing body of evidence that mmWave DE can induce severe biological alterations at the cellular level, particularly affecting cell viability and membrane integrity. Increased LDH leakage in the center group relative to both the penumbra and control groups is a reliable indicator of extensive membrane damage, which is a precursor to necrotic cell death (22). This pattern suggests that direct mmWave DE exposure compromises cellular membranes, leading to cell death, which corroborates findings from Zymantiene et al. (2020), who showed that RF exposure caused similar loss of membrane integrity in cortical neurons (16). Additionally, these results are supported by recent literature that links increased LDH release with various forms of cell stress and death under electromagnetic exposure as discussed by Pall (2022), who explores the broader biological implications of mmWave exposures (7). Given the expanding deployment of mmWave technology in various sectors, including telecommunications and defense, understanding the biological impacts of such exposures is crucial for public health and safety (7, 24, 25, 26). Accordingly, it is essential to elucidate the underlying molecular mechanisms driving this cellular membrane damage and cell death, both of which have been observed as effects of RF-EMR on cellular structures.

To achieve this aim, the expression levels of proteins associated with cell membrane integrity were evaluated in this study. Critical structural proteins such as αII-Spectrin and Vimentin play essential roles in maintaining cellular integrity and function (27, 28). The exposure of N2A cells to mmWave DE has significant implications for cellular integrity, as evidenced by alterations in the expression of αII-Spectrin and Vimentin (27, 29, 30, 31). The analysis through Western blotting demonstrated variable expression levels of these proteins, reflecting the differential impact of DE exposure on cell structure, particularly in areas directly under the DE source compared to more peripheral regions. The differential expression patterns observed between the center and penumbra groups further illustrate the spatial impact of DE exposure, with cells closer to the DE source experiencing more pronounced protein degradation.

According to previous studies, this gradient effect highlights the need for spatial considerations in evaluating the biological impacts of DE exposure, as cells at varying distances from the source may experience different levels of stress and damage, potentially leading to varied biological outcomes (20, 32, 33, 34, 35).

αII-Spectrin, a cytoskeletal protein, plays a pivotal role in maintaining cellular structure and membrane integrity (27, 29). Its breakdown products, particularly at 120 kDa, significantly increased in cells from both the center and penumbra groups exposed to DE, indicating enhanced apoptotic-induced caspase proteolysis (36). It has been reported that RF-EMR induces changes in protein expression, leading to the production of reactive oxygen species (ROS) and caspase-3-dependent apoptosis (37). There are many reports of ROS increase after RF exposure in the brain which in turn give rise to a cascade of molecular events involving protein kinase C (PKC) and finally the pro-apoptotic enzyme caspase 3 (38, 39, 40). PKC plays a role in the caspase-3-mediated proteolysis of αII-Spectrin, a cytoskeletal protein involved in maintaining cell structure and integrity (41). This suggests that mmWave DE may disrupt cellular architecture, leading to potential cellular dysfunction or death (36, 42). These findings align with those reported in studies examining RF-EMR exposure, where similar disruptions in cell membrane proteins were observed and were associated with adverse cellular outcomes (43).

Vimentin, a key intermediate filament protein essential for maintaining cell integrity and facilitating intracellular signaling (28, 44), exhibited a significant increase in expression following mmWave DE exposure, particularly in the center and penumbra regions. This increase suggests that the cells underwent substantial structural stress in response to DE exposure (45). The Vimentin breakdown product (VBDP) at 45 kDa, which was notably elevated in the center group, further supports the notion of enhanced protein degradation and structural remodeling, indicative of cellular damage or repair mechanisms triggered by the environmental stressor (31). The upregulation and breakdown of Vimentin in this study suggest an active response to intermediate filament destabilization, a common feature of cellular stress responses (46, 47, 48). Under stress, Vimentin undergoes calpain-mediated proteolysis, a calcium-dependent process triggered by increased intracellular calcium levels. This pathway selectively degrades Vimentin while sparing actin and tubulin, enabling targeted cytoskeletal remodeling (49). These mmWave DE exposure-induced alterations in Vimentin expression, which caused neuronal damage, are consistent with findings from previous studies, such as those by Zymantiene et al. (2020), who reported similar changes in essential proteins under exposure to EMR. In their work, EMR exposure led to neuronal shrinkage and vacuolization, both indicative of cytoskeletal rearrangements and cellular dysfunction (16).

In the context of mmWave DE exposure, these findings imply that Vimentin, along with its breakdown products, may play a critical role in mediating cellular responses to environmental stress, possibly by contributing to cytoskeletal reorganization or repair mechanisms. The increased Vimentin turnover observed here may also be linked to disruptions in the neuronal cytoskeleton, which is crucial for maintaining cell shape, signaling pathways, and overall cellular homeostasis (28, 31, 44, 45). These alterations could reflect early signs of cellular injury, indicating the need for further investigation into how Vimentin and similar structural proteins might serve as biomarkers for mmWave DE-induced cellular damage. This research will be crucial for understanding the molecular mechanisms underlying the neurological impacts of DE exposure and developing protective strategies to mitigate its effects. This study emphasizes the importance of monitoring changes in structural proteins as biomarkers for assessing the impact of mmWave DE on cellular health. The results suggest that even short-term exposure to DE can lead to significant alterations in cellular architecture, which could have long-term consequences for cell function and viability. Future studies should continue to explore these relationships, particularly in the context of chronic exposure and in vivo systems, to better understand the full scope of health implications associated with emerging DE technologies. Such research is crucial for developing effective protective strategies and safety standards to mitigate the potential risks associated with mmWave DE exposure in both civilian and military contexts.

The mmWave DE exposure induces profound proteomic changes indicative of cytoskeletal disintegration and trafficking disruption in neuronal cells. The induction of CRLS1, a critical enzyme in the synthesis of the mitochondrial phospholipid cardiolipin (50, 51), suggests a cellular response to mitochondrial membrane stress, which aligns with the observed decrease in cell viability (52). The upregulation of KIF5C, a microtubule-associated motor protein involved in organelles and vesicles transport, may suggest a compensatory response to restore disrupted intracellular trafficking induced by irradiation stress. However, in the context of overwhelming damage, this elevation likely reflects dysregulated trafficking, contributing to neuronal process degradation and cellular disorganization (53, 54). Similarly, the upregulation of TOR3A, a member of the AAA + ATPase family, suggests a cellular attempt to restore homeostasis. This may reflect endoplasmic reticulum stress responses to misfolded proteins or disrupted nuclear envelope dynamics, potentially contributing to the vacuolization and abnormal nuclear morphology observed in irradiated cells (55, 56, 57). Concurrently, the downregulation of several critical proteins underscores the collapse of key survival and maintenance pathways. The downregulation of MAPRE1, a microtubule plus-end tracking protein, impairs microtubule elongation and attachment, contributing directly to cytoskeletal collapse and loss of cell polarity. These alterations lead to morphological changes in cell shape and compromise membrane integrity (58, 59, 60, 61). BIN1, a membrane curvature regulator, is also downregulated, impairing the cell’s ability to remodel membranes and disrupt t-tubule structure, thereby making the membrane more susceptible to damage and leakage. This compromise in plasma membrane stability may underlie the observed loss of membrane integrity, fragmented processes, and contribute to cell shrinkage or blebbing, which are consistent with apoptotic morphology (62, 63). Furthermore, HMGN3 downregulation suggests diminished chromatin accessibility and transcriptional stress responses that accelerate apoptotic signaling (64, 65). In contrast, loss of SUB1, a transcriptional coactivator, further impairs global gene expression and reduces protein synthesis, thereby promoting cellular shutdown and death under stress conditions. (66, 67). Collectively, these changes converge on a phenotype of progressive neuronal degeneration, marked by loss of polarity, decreased viability, and structural collapse—a mechanistic signature of mmWave DE-induced cellular injury.

Sub-lethal mmWave exposure triggered a concerted nuclear and cytoskeletal stress programme in Penumbra-zone neurons. The upregulation of these proteins, which are involved in nuclear processes such as mRNA export (POLDIP3) (68), histone deacetylation (SAP18) (69), and immune/apoptotic regulation (PTMA) (70), suggests an active transcriptional and translational reprogramming effort in response to sublethal stress. This contrasts sharply with the signature of the acute structural collapse in the Center. Loss of Nestin as an intermediate filament protein which is essential for the proliferation and survival of neural progenitor cells and plays a critical role in maintaining cytoskeletal integrity leads to cell-cycle arrest and reduced self-renewal. Dysregulated Nestin expression may destabilize cytoskeletal filament organization especially when co-assembled with Vimentin potentially contributing to morphological changes (71, 72, 73, 74). ACTR3B, an ATP-binding subunit of the Arp2/3 complex, plays a critical role in nucleating branched actin filaments and maintaining cytoskeletal architecture. The Arp2/3 complex contributes to cell-cell adhesion, cell polarity, and tight junction maintenance. Under conditions of cellular stress, dysregulated Arp2/3 activity can disrupt actin network assembly and lead to morphological decay, including structural collapse and membrane compromise (75, 76, 77). In stark contrast, key RNA-processing, trafficking and repair factors are suppressed, shifting the balance toward degeneration. Downregulation of the core spliceosomal protein SNRPF disrupts normal pre-mRNA splicing, leading to aberrant transcripts and reduced protein synthesis, which can impair cellular repair mechanisms (78). Loss of SUB1, a coactivator of RNA polymerase II, leads to global transcriptional repression, resulting in reduced metabolic activity, impaired repair capacity, cellular shutdown, and decreased cell viability (66, 67). Downregulation of VAMP4, a key regulator of vesicular transport and calcium-regulated exocytosis, suggests impaired membrane trafficking and granule maturation, leading to secretory dysfunction and membrane instability (79, 80, 81). Downregulation of RAB13 disrupts both the structure and function of tight junctions. Disruption of junctional proteins compromises barrier function and cell polarity, often leading to cytotoxic outcomes that encompass membrane blebbing, LDH release and eventually actin disruption- induced cell death (82, 83). Depletion of UBE2N, a key E2 ubiquitin-conjugating enzyme that catalyzes non-proteolytic K63-linked ubiquitin chains, impairs DNA damage signaling and repair pathways, thereby compromising cell survival mechanisms and increasing susceptibility to stress-induced apoptosis (84, 85). Collectively, these proteomic shifts outline a two-pronged mechanism: a transient nuclear-cytoskeletal repair attempt that is quickly undermined by failures in mRNA processing, membrane trafficking and DNA repair, culminating in reduced viability and progressive structural collapse in Penumbra neurons.

### Limitations of the study

Despite the novel insights provided, this study has several limitations that must be acknowledged. First, the study utilizes an immortalized mouse neuroblastoma cell line (N2A). While a valid and widely used model for initial mechanistic investigation, these are cancer-derived cells with altered proliferation rates and signaling pathways compared to primary post-mitotic neurons. Therefore, the findings require rigorous validation in more physiologically relevant models, such as primary rodent cortical neurons, human iPSC-derived neurons, brain organoids, or ultimately, *in vivo* animal models, to understand the effects in the context of a complex tissue environment.

Second, the results are specific to a single carrier frequency (34.14 GHz) and a particular exposure regimen (intermittent, 48h). The biological effects of RF-EMR are known to be highly dependent on a range of physical parameters, including frequency, modulation, polarization, and power density. Extrapolation of these findings to other frequencies within the mmWave spectrum (e.g., other 5G bands) or to different exposure scenarios (e.g., chronic low-level exposure) must be done with caution. Future studies are needed to determine if the observed “triad of collapse” is a general response to high-intensity RF-EMR or a frequency-specific phenomenon.

Finally, while extensive care was taken to control for bulk heating of the culture medium, it is impossible to completely rule out the contribution of microscopic thermal gradients or “hot spots” at the subcellular or membrane level that could contribute to the initiation of the observed stress responses (86). The precise and organized nature of the downstream proteomic response argues strongly against simple, uniform heating as the sole driver of the observed phenotype. However, the initial trigger may indeed involve localized energy deposition that initiates the complex biological cascade we have described.

### Future directions

The mechanistic model proposed herein provides a clear roadmap for future investigation. The immediate priority is to validate the causal roles of the key proteins identified in our “triad of collapse.” Using siRNA or CRISPR-mediated gene silencing to knock down CRLS1, BIN1, and MAPRE1 in N2A cells before mmWave exposure would directly test whether the absence of these proteins sensitizes cells to the insult. Conversely, overexpressing these proteins could test for a protective effect. These molecular interventions should be coupled with direct functional assays, such as measuring mitochondrial membrane potential (e.g., using TMRM/JC-1 dyes), ROS production (e.g., using DCFDA/MitoSOX), cellular ATP levels, and real-time caspase-3/7 activity to confirm the proposed sequence of events from mitochondrial crisis to apoptotic execution.

The ultimate goal is to translate these *in vitro* findings into a physiological context. Future studies should employ *in vivo* exposure models in rodents, applying similar mmWave DE paradigms. Brain tissue from exposed animals could then be analyzed via proteomics to determine if the same proteomic signature of neurotoxicity (e.g., dysregulation of Crls1, Bin1, Mapre1) is recapitulated. These molecular analyses should be correlated with comprehensive behavioral testing to assess for deficits in learning, memory, anxiety, and motor function, thereby linking the molecular damage to functional neurological outcomes. Furthermore, the potential of the most robustly altered proteins or their specific cleavage products (e.g., SBDP120) as non-invasive biomarkers of DE exposure should be explored in biofluids such as plasma or cerebrospinal fluid.

## Conclusion

In conclusion, this study leverages unbiased, quantitative proteomics to provide an unprecedentedly detailed molecular portrait of mmWave- DE induced neurotoxicity. We have identified a core pathogenic axis involving mitochondrial damage, endo-lysosomal dysfunction, and cytoskeletal collapse that drives a spatially-graded, dose-dependent cell death. These findings not only establish a robust mechanistic framework for understanding the bio-safety of emerging mmWave technologies but also provide a plausible molecular basis for unexplained neurological syndromes and highlight novel and concerning parallels with the pathology of neurodegenerative disease. This work underscores the critical need for further mechanism-based research to establish evidence-based safety guidelines for the ever-expanding spectrum of radiofrequency technologies in our environment.

## Supplementary Material

This is a list of supplementary files associated with this preprint. Click to download.

- SupplementaryTable.xlsx
- SupplementaryFigure1.png
- SupplementaryFigure2.png
- GraphicalAbstract.png
- SupplementaryInformation.docx

## Data Availability

The proteomics raw data used to support the findings of this study are available in the Data Dryad database and hyperlink to dataset in http://datadryad.org/share/urjsczcC2ijfs5aQ19WvGGfmBe-N3yx0AaNzoDUHBWw

## Abbreviations

%: Percentage
5G: Fifth-Generation
ACN: Acetonitrile
AP: Alkaline phosphatase
ATCC: American Type Culture Collection
BP: Biological Process
BSA: Bovine serum albumin
CE: Collision energy
CID: Collision-induced dissociation
cm: Centimeter
CO_2_: Carbon dioxide
CRISPR: Clustered regularly interspaced short palindromic repeats
DCFDA: Dichlorodihydrofluorescein diacetate
DE: Directed energy
DEPs: Differentially expressed proteins
DEW: Directed energy weapon
DMEM: Dulbecco’s Modified Eagle’s Medium
DTT: Dithiothreitol
EMF: Electromagnetic fields
EMR: Electromagnetic radiation
ER: Endoplasmic reticulum
FA: Formic acid
FBS: Fetal bovine serum
FC: Fold change
FDR: False discovery rate
GHz: Gigahertz
GO: Gene Ontology
GSM: Global System for Mobile Communications
h: Hour(s)
IAA: Iodoacetamide
IPA: Ingenuity Pathway Analysis
kDa: kilodalton
LC-ESI-MS/MS: Liquid chromatography-electrospray ionization-tandem mass spectrometry
LC-MS/MS: Liquid chromatography-mass spectrometry/mass spectrometry
LDH: Lactate dehydrogenase
LFQ: Label-Free Quantification
LTQ: Linear trap quadrupole
m/z: mass-to-charge ratio
M: Molar
mAb: monoclonal antibody
MF: Molecular Function
mg: Milligram
MHz: Megahertz
mL: Milliliter
mM: Millimolar
mmWave: Millimeter-wave
MRI: magnetic resonance imaging
mRNA: Messenger ribonucleic acid
MTT: 3-(4,5-Dimethylthiazol-2-yl)-2,5-diphenyltetrazolium bromide
mW/cm^2^: Milliwatts per square centimeter
MWM: Molecular weight markers
N2A: Mouse neuroblastoma
NaCl: Sodium chloride
NAD: Nicotinamide adenine dinucleotide
NADH: Nicotinamide adenine dinucleotide (reduced)
nL/min: Nanoliter per minute
nm: Nanometer
OD: Optical density
PBS: Phosphate-buffered saline
PC1: Principal Component 1
PCA: Principal component analysis
pH: potential of hydrogen
PKC: Protein kinase C
PSMs: Peptide-spectrum matches
PVDF: Polyvinylidene difluoride
RF-EMR: Radio frequency-electromagnetic radiation
RF: Radio frequency
RNA: Ribonucleic acid
ROS: Reactive oxygen species
rpm: Revolutions per minute
SBDP: αII-Spectrin Breakdown Product
SDS-HCl: Sodium dodecyl sulfate-hydrochloric acid
SDS-PAGE: Sodium dodecyl-sulfate polyacrylamide gel electrophoresis
SEM: Standard error of the mean
siRNA: small interfering RNA
TBST: Tris-Buffered Saline with Tween 20
TMRM/JC-1: Tetramethylrhodamine methyl ester, and JC-1
UPR: Unfolded protein response
VBDP: Vimentin Breakdown Product
°C: Degrees Celsius
μL: Microliter
μg: Microgram
μm: Micrometer
